# P04.57. Music therapy in the treatment of cancer patients: a systematic review

**DOI:** 10.1186/1472-6882-12-S1-P327

**Published:** 2012-06-12

**Authors:** T Ostermann, C Boyde, U Linden

**Affiliations:** 1 University of Witten/Herdecke, Herdecke, Germany; 2 Department of Music Therapy, Community Hospital Herdecke, Herdecke, Germany

## Purpose

Creative therapies like painting, speech therapy, healing eurythmic dance therapy and music therapy are frequently used in the treatment of cancer patients. Particularly in the last decade, several studies have concentrated on the investigation of effects of music therapy in cancer patients.

## Methods

The following databases were used to find studies of music therapy in oncology: AMED, CAIRSS, EMBASE, MEDLINE, PsychINFO, and PSYNDEX. The search terms were [“Study OR Trial” AND “Music Therapy” AND “Cancer or Oncology”]. Included studies were analysed with respect to their study design and quality, setting and interventions including date of publication, indications, patients and main outcomes.

## Results

We found a total of 12 clinical studies conducted between 2001 and 2011 including a total of 922 patients. Eight studies had a randomized controlled design and four studies were conducted in the field of pedeatric oncology. Both type and grading of cancer were heterogenous thoughout all studies. Active music making (n=7) as well as listening programs (n=5) were mostly enrolled after surgery or chemotherapy to improve the patient’s situation. Studies reported on short term improvements in patient’s mood, relaxation, lowering exhaution and anxiety as well as in coping with the disease and cancer related pain.

## Conclusion

The use of music therapy in the integrative treatment of cancer patients is a therapeutic option, whose salutogenetic potential is shown in many case studies. Study results however did not draw a conclusive picture on the overall effect of music therapy. Research therefore should investigate the underlying mechanisms to come to a more conclusive hypothesis.

